# The story of memory and executive functions in obsessive-compulsive disorder: a case-control study

**DOI:** 10.47626/2237-6089-2021-0243

**Published:** 2022-09-13

**Authors:** Sajedeh Hamidian, Abbas Pourshahbaz, Esmaeil Shahsavand Ananloo, Behrooz Dolatshahi, Mina Ohadi, Mohammadreza Davoudi

**Affiliations:** 1 Department of Clinical Psychology USWR Tehran Iran Department of Clinical Psychology, University of Social Welfare and Rehabilitation Sciences (USWR), Tehran, Iran.; 2 Imam Khomeini Hospital Complex School of Medicine Tehran University of Medical Sciences Tehran Iran Department of Psychosomatic, Imam Khomeini Hospital Complex, School of Medicine, Tehran University of Medical Sciences (TUMS), Tehran, Iran.; 3 Iranian Research Center on Aging University of Social Welfare and Rehabilitation Sciences Tehran Iran Iranian Research Center on Aging, University of Social Welfare and Rehabilitation Sciences, Tehran, Iran.

**Keywords:** Executive functions, memory, neuropsychology, obsessive-compulsive disorder, Wechsler memory scale

## Abstract

**Objective:**

Neuropsychological findings in obsessive-compulsive disorder (OCD) are mainly clustered around the role of memory and executive functions. However, outcomes vary across different OCD populations. In addition, the extent to which each of these factors can distinguish patients with OCD (PwOCD) from healthy individuals remains uncertain and attracts great attention. The present study aims to investigate the above issues.

**Method:**

This was a cross-sectional study of 182 individuals (90 PwOCD and 92 matched healthy controls). After screening for inclusion and exclusion criteria, the participants were administered neuropsychological tests including, the Wechsler Memory Scale-III (WMS-III), the Wisconsin Card Sorting Test (WCST), and the Stroop Color-Word Test (SCWT). Data were analyzed to test the study hypotheses using comparison of means and regression analysis methods.

**Results:**

The results showed that PwOCD had poorer performance than the control group in Immediate Memory, General Memory, and Working Memory and also according to response inhibition indexes. The results also showed that General Memory and Reaction Time2 from the SCWT index could be predictive variables for discriminating between PwOCD and controls.

**Conclusion:**

The findings of this study support the prior assumptions that PwOCD would have impaired memory dimensions and response inhibition, but did not support worse set-shifting performance. We also present an initial model for the predictive role of these neuropsychological variables in discriminating OCD from healthy individuals and increasing diagnostic accuracy.

## Introduction

Obsessions are repetitive thoughts, urges, or images that are intrusive and unwanted, and in most people cause anxiety or distress. Compulsions are repetitive mental or overt acts that are experienced as being urge-driven either in response to an obsession or according to a rule that must be applied decisively and are aimed at preventing or reducing anxiety or distress or preventing a feared event happening.^1^

The diagnostic and statistical manual of mental disorders, 5th edition (DSM-5), states that these symptoms are not specific to obsessive-compulsive disorder (OCD) and at least 14 other disorders have symptoms broadly meeting these criteria such as body dysmorphic, trichotillomania, skin picking, generalized anxiety, depressive, and addictive disorders.^2^ Non-specific symptoms are not exclusive to OCD, but are common to almost all psychiatric disorders. Due to these not very precise boundaries, the National Institute of Mental Health (NIMH) has launched its own Research Domain Criteria (RDoC), which suggest a framework to define clinical disorders based on cognitive, physiological, molecular, and genetic levels.^3^ Based on this view, a promising approach to clarify the neurobiology of psychiatric disorders is to seek structures that can be placed somewhere between low-level causative agents and phenotypic manifestations of the disorder, called the endophenotype.^4 , 5^

A trait must have several characteristics to be considered as an endophenotype; it must be associated with the disease, heritable, state-independent, and found in unaffected family members of a patient more frequent than in the general population.^6^ Memory is one of the cognitive domains that have attracted the most research attention. Patients with OCD (PwOCD) showed shortcomings in short-term memory, working memory, and visual-spatial abilities.^7^ However, findings are still remarkably inconsistent. A group of studies have found no significant impairment in memory dimensions, while the others, which constitute a larger proportion of the studies, emphasize frequent memory impairments in PwOCD. Among the second group, identification of the memory aspects with the most disturbances in OCD is still controversial. Although, non-verbal memory such as long-term and immediate visuospatial memory problems have been consistently demonstrated in OCD, the findings related to verbal memory performance are relatively inconsistent.^8 , 9^ Some researchers reported intact working and declarative verbal memory and some others have reported difficulties in recalling verbal episodic material.^7 , 10 , 11^

A group of studies have suggested that defects in memorizing and recalling are due to defects in the organization of information and emphasize the role of executive functions in memory impairments in OCD.^12 , 13^ Disturbances in several features of executive functions have been proposed as endophenotypes of OCD.^14^ Impaired performance in working memory, attentional set-shifting ability that underlies cognitive flexibility, and response inhibition have been repeatedly reported by many studies, even after controlling for age, gender, and education.^9 , 14 - 18^ Set shifting and response inhibition are assumed to be responsible for perseveration and repetitive behaviors. Defects in these areas could be the basis of impulsive and compulsive acts common in patients with OCD.^19 , 20^

A meta-analysis of studies of cognitive functioning in OCD reported large effect sizes for nonverbal and visuospatial memory. In contrast, set-shifting, cognitive inhibition, and verbal memory had medium or small-to-medium effect size. Working memory results are more inconsistent, varying from small to large effect sizes.^7 , 21 , 22^

The abovementioned studies in this area were mostly performed on smaller samples using simpler tools. So, in this study with a relatively large sample and using more comprehensive tools we will assess substantial areas of memory and executive functions in a large group of patients with obsessive-compulsive disorder. Moreover, by analyzing mediating neuropsychological features, we will estimate each variable’s contribution to developing OCD and the extent to which they can discriminate PwOCD from healthy individuals. Although exact determination of this pattern is very complicated because of the high rates of comorbidity and dimensional distribution of traits in psychiatric disorders, we attempt to discover a preliminary model among a group of obsessive-compulsive patients.

## Methods and materials

### Participants and procedure

**Figure 1:**
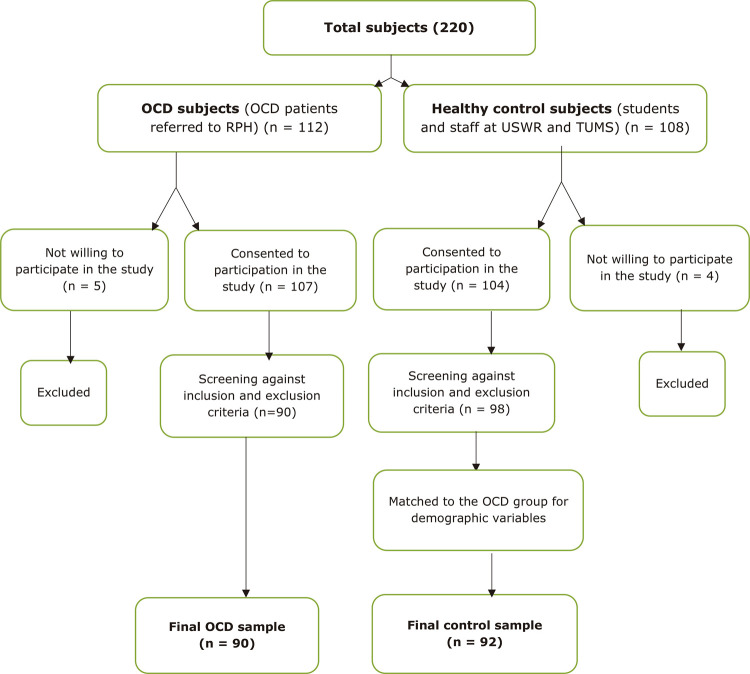
Flowchart diagram of sampling and processing. OCD = obsessive-compulsive disorder; RPH = Roozbeh Psychiatric Hospital; TUMS = Tehran University of Medical Sciences; USWR = University of Social Welfare and Rehabilitation Sciences.

The study participants were 182 individuals, comprising 90 patients with obsessive-compulsive disorder and 92 healthy controls. The sampling process is described in Figure 1 . The case group comprised people who had been referred to the outpatient clinic of Roozbeh Psychiatric Hospital (RPH), affiliated to Tehran University of Medical Sciences (TUMS), and were diagnosed with obsessive-compulsive disorder based on DSM-5 criteria. Patients were enrolled on the study provided they fulfilled the following: Yale-Brown Obsessive Compulsive Scale (Y-BOCS) severity greater than 16, aged between 18 and 60 years, having at least five years of education, and diagnosed with obsessive-compulsive disorder based on DSM-5 criteria as the primary diagnosis. Patients were excluded from the study if they had current or lifetime history of psychotic disorders such as bipolar and schizophrenia disorder or severe depression (scores higher than 29 on the Beck Depression Inventory 2), or had one of the obsessive-compulsive and related disorders category as main diagnosis and a drug and/or alcohol dependency. Furthermore, people with diseases that affect cognitive functions such as dementia, history of head trauma, epilepsy or surgery in the past two weeks, or history of receiving electroconvulsive therapy (ECT) in the last 6 months were excluded. All of the patients were taking psychotropic medications for treatment of OCD. The healthy control group were selected from among university students and staff at TUMS and the University of Social Welfare and Rehabilitation Sciences (USWR) by purposeful sampling using the exclusion and inclusion criteria. They were matched to the case group for age, gender, and years of education and had no history of current or previous psychiatric or neurological disorders. They also should not be taking medications that affect memory and consciousness.

Since the measures were sensitive to stimulus interference and distractibility, they were administered in a quiet and distraction-free room in the Department of Genomic Psychiatry and Behavioral Genetics at Roozbeh Hospital. Some of the subjects in the case and control groups did not complete all the tests due to fatigue, hurry, or unwillingness. Neuropsychological data on some measures are therefore missing for a few participants.

### Ethical considerations

Participants were given a full explanation about the project and were enrolled after signing informed consent. Participants were assured that their identifiable information would remain confidential, ensuring anonymity. The present study is part of a project that has been approved at the USWR, with Ethics Code Ir.uswr.rec.1396.216. This project was administered in accordance with the General Ethic Codes for research approved by the USWR Ethics Committee.

### Measures

#### Wechsler Memory Scale-III (WMS-III)

The Wechsler Memory Scale-III (WMS-III) is the third edition of the original version of the Wechsler Memory Scale (WMS) and provides a comprehensive assessment of different memory functions. It includes 11 primary and 7 supplementary subtests. There are three main indexes obtained from the primary subtests, Immediate Memory (also known as learning), General Memory (delayed memory), and Working Memory. The Immediate Memory score is the sum of scores for immediate recall of Logical Memory 1, Faces 1, Verbal Paired Associations 1, and Family Pictures 1. The General Memory index is the sum of delayed scores for the same four subtests plus the Auditory Recognition Delayed score. The Working Memory index is composed of Letter ± Number Sequencing and Spatial Span subtests. The Persian version of WMS-III has shown suitable psychometric properties.^23^

#### Stroop Color-Word Test (SCWT)

A computerized version of the Stroop Color-Word Test was used. This test consists of two phases, a preliminary and a main phase. In the preliminary phase, the subject is asked to respond by pressing the key corresponding to the color of a circle shown on the screen. The goal of this step is just to recognize the colors and placement of keys on the keyboard and it does not affect the final result. In the main phase, 48 congruent colored words (the word color was identical with the meaning of the word) and 48 incongruent colored words (the color of the word was not the same as the word meaning) were displayed for 2000 milliseconds at 800 millisecond intervals. The subject’s task was to specify the color of the word, regardless of the word meaning. The test results for the two phases of congruent and incongruent color-word combinations are presented in the results section with numbers of 1 and 2, respectively.^24^

#### Wisconsin Card Sorting Test (WCST)

A computerized version of the Wisconsin Card Sorting Test that has been normed for the Iranian population was used. This test is mainly used to measure frontal cortex executive functions and is known as an index of set-shifting ability and cognitive persistence. The subject was presented with a set of 64 cards; each of them represents one to four symbols in four colors.^25^

#### The Yale-Brown Obsessive-Compulsive Scale (Y-BOCS)

The Yale-Brown obsessive-compulsive scale (Y-BOCS) is a semi-structured interview for assessing the severity of obsessive and compulsive symptoms. This scale consists of 10 questions that measure the domains of distress, frequency, intervention, resistance, and symptoms control in two parts, one for obsessions and the other for compulsions. A cut-off point of 9 on the Persian version of Y-BOCS has been found to differentiate patients from healthy controls. It has been reported that the Persian version of the scale has good test-retest reliability, reliability, and validity.^26^

#### Beck Depression Inventory-2 (BDI-II)

This inventory was administered to evaluate the severity of depressive symptoms. The items are scored from 0-3, ranging from absence of a specific symptom to presence of the symptom to the highest degree. Furthermore, Mohammadkhani and Dobson reported that the Persian version of this questionnaire had a reliability coefficient of 0.913 for its 21 items.^27^

## Statistical analysis

Data were analyzed using SPSS version 22 software. The chi-square test was used to compare nominal demographic variables between the OCD and normal subjects. The independent *t* test was used to compare continuous demographic variables and clinical and neuropsychological variables with normal distribution and equality of variances. The Mann-Whitney U test was used for neuropsychological variables without normal distribution. In addition, Pearson correlation coefficients were used to determine correlations between neuropsychological variables. Finally, logistic regression (LR) was used to develop a model for discriminating between the OCD and control groups.

## Results

### Demographic and clinical characteristics

Table 1 lists the demographic and clinical features of the case and control groups. As shown in Table 1 , there were no statistically significant differences between the two groups in age, gender, years of education, or handedness. Mean Y-BOCS scores for the cases were 12.7±3 for obsessions and 12.5±3.7 for compulsions and the mean total score was 25.3±5.4. Furthermore, the mean score for depression in the case group was 18.7±11.9, compared to 7.4±7.8 for the control group. These data are presented in detail in Table 1 .

**Table 1:** Demographic and clinical data in the OCD and control groups

	OCD group (n = 90)		Control group (n = 92)		Chi-square	p
Gender (M/F)	34 (37%) M	56 (62%) F	33 (35%) M	59 (64%) F	0.071	0.79
Handedness (R/L)	78 (86%) R	12 (13%) L	85 (92%) R	7 (7%) L	2.295	0.13
	
	**Mean**	**SD**	**Mean**	**SD**	***t* test**	**p**
	
Age	33.5	9.3	32.5	9.6	-0.74	0.46
Years of education	13.4	9.1	14.1	3.2	1.5	0.10
Y-BOCS-Obsession	12.7	3	-	-		
Y-BOCS-Compulsion	12.5	3.7	-	-		
Y-BOCS-Total	25.3	5.4	-	-		
BDI-II	18.7	11.9	7.4	7.8	-7.3	<0.001

BDI-II = Beck Depression Inventory-2; F = female; L = left handed; M = male; OCD = obsessive-compulsive disorder; R = right handed; SD = standard deviation; Y-BOCS = Yale-Brown Obsessive Compulsive Scale.

### Neuropsychological assessment

#### Between-group comparison analysis

The results of the mean comparisons, standard deviation, and *t* test or Mann-Whitney U test between the two groups are shown in Table 2 .

**Table 2:** Results of comparisons of mean WMS-III, SCWT, and WCST variables between the OCD and control groups

n = case/control	Case		Control		*t*	p

	Mean	SD	Mean	SD		
Memory (90/92)						
Audio immediate	56.2	17.2	66.6	15.2	4.32	< 0.001
Visual immediate	65.8	16.3	73.92	14.4	3.51	< 0.001
Immediate memory	122.1	29.1	140	26.9	4.3	< 0.001
Audio delayed	30.3	10.3	35.9	8	4.06	< 0.001
Visual delayed	63.3	16.6	72.7	14.5	4.07	< 0.001
Audio recognition delayed	49.1	5.6	49.8	2.9	1.07	0.28
General memory	156.3	28.7	174.1	24.5	4.48	< 0.001
Working memory	23.2	6.2	25.4	5.3	2.57	0.011
Stroop (85/88)						
Error 1	0.52	1.4	0.44	0.8	3595	0.57
No response 1	2.08	4.06	0.68	1.6	2635.5	0.000
True 1	45.38	5	46.8	1.8	3123	0.04
Reaction time 1	1102.1	213.5	980.6	145.3	2449.5	< 0.001
Error 2	3.61	8.6	1.87	4.8	3247	0.11
No response 2	3.45	5.9	1.06	1.9	2705.5	< 0.001
True 2	40.92	11.4	44.96	5.6	2977	0.01
Reaction time 2	1169.9	218.2	1040.7	154.2	2393.5	< 0.001
Test result	4.45	9.2	1.84	5.2	3069	0.03
Result time	68.96	79.4	60	39.8	3533	0.53
Wisconsin (86/88)						
Categories completed	2.67	1.8	3.15	1.8	3196.5	0.07
Perseveration errors	10.59	8.3	8.82	6.3	3402	0.24
Total number correct	30.18	8.2	32.3	8	3202.5	0.08
Test time	343.98	131.4	268.2	65.9	2304.5	< 0.001
Trylevone*	18.73	14.6	15.71	12.5	3408	0.25
Percentcon^†^	50.31	47.3	64.9	44.3	3177.5	0.04

OCD = obsessive-compulsive disorder; SCWT = Stroop Color-Word Test; SD = standard deviation; WCST = Wisconsin Card Sorting Test; WMS-III = Wechsler Memory Scale-III.

* Trylevone = trials to complete first category.

^†^ Percentcon = percentage of Conceptual Level Responses.

**Wechsler Memory Scale-III (WMS-III).** As shown in Table 2 , the OCD group showed a significantly poorer performance compared to the control group in Audio Immediate (p < 0.001), Visual Immediate (p < 0.001), Audio Delayed (p < 0.001), and Visual Delayed (p < 0.001) and in the three main indexes of Immediate Memory (p < 0.001), General Memory (p < 0.001) and Working Memory (p = 0.011). The only index which did not show a significant statistical difference between the groups was Audio Recognition Delayed (p = 0.28).

**Stroop Color-Word Test (SCWT).** Since the condition of normal distribution and equality of variances was not met, we used Mann-Whitney U to compare OCD and control groups’ mean scores. As shown in Table 2 , there were significant differences between the OCD and normal subjects for true responses (True 1, p = 0.048; True 2, p < 0.001), No Responses (NoR 1, p < 0.001; NoR 2, p < 0.001), and Reaction Time to the stimulus (ReT 1, p < 0.001; ReT 2, p < 0.001) in both phases, as well as for the interference index (Test Result, p = 0.039).

**Wisconsin Card Sorting Test (WCST).** In the present study, the OCD group only showed poorer performance in two indexes, the time test (p < 0.001) and the percentage of conceptual level responses (Percentcon, p = 0.04). Simultaneously, no significant differences were observed in main indices like the number of categories completed and perseveration errors.

#### Logistic regression (LR)

Logistic regression was employed to develop a hypothesized model for the role of each variable in distinguishing the OCD group from the healthy group. As the results of Table 3 show, among the six variables entered into the model, “Response time” from the SCWT and “General Memory” from the WMS-III can significantly predict discrimination. The Hosmer and Lemeshow test (chi-square = 11.2, p = 0.17) showed the model had appropriate fit.

**Table 3:** - Logistic regression analysis for discriminating OCD and healthy groups using General Memory, Working Memory, No Response 2, Reaction Time 2, Test Result, and Percentcon variables

Predictor	B	S.E.B	Wald	OR	p
Reaction time 2	0.003	0.001	7.507	1.003	.006
General memory	-.016	.007	5.259	.984	.02
Constant	-.358	1.891	.036	.699	.85

B = unstandardized beta; OCD = obsessive-compulsive disorder; S.E.B = standard error for unstandardized beta; OR = odds ratio.

The results of statistical analysis showed that the model has an accuracy rate of 67% (Classification accuracy = 67.6) and can account for 18% (Nagelkerke R Square = 0.18) of differentiation between the two groups.

## Discussion

This study’s results support the assumptions about the impaired performance of PwOCD in memory dimensions and in response inhibition compared to the healthy controls. The other assumption about the poorer performance of PwOCD in set shifting was not supported. As the results of memory components indicate, PwOCD showed poorer performance in three domains, Immediate Memory, General Memory (which is an index of episodic memory), and Working Memory. Although the literature about memory functioning chiefly emphasizes impairments in nonverbal rather than verbal memory,^7 , 22^ our findings showed both verbal and nonverbal deficits in PwOCD. In terms of auditory memory, which is mainly supposed to measure verbal material, our findings showed PwOCD had weaker performance, which is in line with studies by Segalas et al. and Rajender et al.^28 , 29^ The flawed verbal memory could be attributed to organizational and semantic clustering strategies that patients use during encoding and learning.^30 , 31^ Regarding nonverbal memory, the Rey-Osterrieth Complex Figure Test (RCFT) was the tool most used to evaluate visual memory in previous studies and the results consistently document the poorer performance of PwOCD.^18 , 32 , 33^ In the present study, despite using a different task to measure nonverbal memory (faces and family pictures from the WMS-III), the same results were observed. Savage et al. explain the defects in nonverbal memory according to difficulties PwOCD have in using organizational strategies.^34^

Working memory was another area that showed impairment in PwOCD. Previous findings about working memory are not consistent. Snyder et al. and Harkin et al. reported poorer performance in working memory, whereas some other studies found no defective working memory in OCD.^13 , 35 , 36^ To explain the contradiction observed, Martoni et al. suggested that working memory in PwOCD gets more problematic as the task becomes more difficult and complicated. Because of the greater need to contain irrelevant information in mind and memory management strategies, PwOCD encounter serious problems with blocking irrelevant information and processing relevant data. In this situation, working memory was more affected by executive functions.^37^ Therefore, since the current study’s working memory task was letter-number sequencing and spatial span, which is considered a complex task, it was expected that PwOCD would have greater difficulty with it.

The SCWT results indicated significantly poorer performance among the PwOCD in selective attention and response inhibition, as inferred by the “Test Result” score. In the incongruent phase of the SCWT, the number of true responses and unanswered responses were significantly reduced relative to the congruent phase. This suggests the inability of PwOCD to manage the stimulus when required to focus on two stimuli. In other words, when the meaning and color of the words are incongruous, they have more trouble in inhibiting the meaning of the word in order to react to its color. SCWT tasks can result in three types of responses: true, error, or unanswered. Interestingly, the pattern of PwOCD’s performance showed that although they had a lower true response rate, they did not compensate it with more errors, whilst their unanswered responses increase compared to the control group. This is congruent with the hesitation and slowness which is usually observed among the clinical features of OCD.

The results for the model developed to address the predictive role of executive functions and memory in discriminating the PwOCD from the healthy group indicated a significant role of General Memory and “Reaction Time 2” from the SCWT, which means an altered pattern of memory and response inhibition in PwOCD. These two variables account for 18% of discrimination between the two groups. Furthermore, as per the model, the significant role of “Reaction Time 2” without a significant “Test Result” could suggest that caution should be exercised in considering executive functions to be the factor that differentiates the two groups in this study. Rather, this differentiation seems to be better explained by the slowness and increased reaction time that affect PwOCDs’ performance in many domains.^38^ Although the idea of developing a model of the contribution of different neuropsychological features in OCD demands more detailed study designs, the prospect of using neuropsychological variables seems to be highly advantageous for diagnostic accuracy.

With regard to the WCST test, the results for specific indexes such as “the number of categories completed” and “perseverative errors” were similar in both groups. Previous findings in this regard have been inconsistent; some showing comparable performances in PwOCD and healthy subjects and others detecting significant differences.^39 , 40^

## Conclusion

The results of this study constitute support for the hypothesis about altered neuropsychological features in PwOCD compared to the healthy subjects. The areas identified include impairments in immediate, episodic, and working memory. In terms of executive functions, the study found significant disturbances in response inhibition but not in set shifting in PwOCD. The model presented was constructed to look for predictive factors that discriminate between the two groups and detected General Memory and Reaction Time in the incongruent stimulus condition as predictive variables. According to the model, none of the executive function indexes could perform a predictive discriminating role, despite being significantly different between the two groups.

## Limitations and future directions

Since all of the PwOCD in this study have been on medication for years, cognitive disturbances could be a predictable side effect. Hence, the memory and executive problems observed here could be attributable to chronic use of medications. Discovering whether the memory deficits in OCD are primary or secondary is the first step, the next steps are to identify their mechanisms of development and maintenance and subsequently their mediating roles in development of clinical manifestations of the disorder. Applying these findings in practice could lead to inclusion of training related to attention, memory, and other cognitive abilities in OCD treatment protocols. Future studies should focus on identifying these defects in the OCD subtypes as well as more specific analyses focusing on memory subsets such as verbal and nonverbal memory.
